# The effects of Transcranial Direct Current Stimulation on food craving and food intake in individuals affected by obesity and overweight: a mini review of the magnitude of the effects

**DOI:** 10.3934/Neuroscience.2022020

**Published:** 2022-08-01

**Authors:** Graziella Orrù, Valentina Cesari, Eleonora Malloggi, Ciro Conversano, Danilo Menicucci, Alessandro Rotondo, Cristina Scarpazza, Laura Marchi, Angelo Gemignani

**Affiliations:** 1 Department of Surgical, Medical and Molecular Pathology and Critical Care Medicine, University of Pisa, via Savi, 10, 56126, Pisa, Italy; 2 Department of Law, Criminal Law, University of Pisa, via Curtatone e Montanara, 15, 56126, Pisa, Italy; 3 Department of General Psychology, University of Padova, Via Venezia 8, Padova, 35131, Italy; 4 IRCCS S Camillo Hospital, Via Alberoni 70, 30126 Venezia, Italy; 5 Padova Neuroscience Centre, University of Padova, Via Giuseppe Orus 2, 35131 Padova, Italy

**Keywords:** food craving, obesity, overweight, food intake regulation, tDCS

## Abstract

Obesity represents one of the wellness diseases concurring to increase the incidence of diabetes, cardiovascular diseases, and cancer. One of the main perpetuating factors of obesity is food craving, which is characterized by an urgent desire to eat a large and various amount of food, regardless of calories requirement or satiety signals, and it might be addressed to the alteration of the dorsolateral prefrontal cortex (DLPFC) activity. Despite most of the gold-standard therapies focus on symptom treatment only, non-invasive brain stimulation techniques such as transcranial direct current stimulation (tDCS) could help treat overeating by modulating specific neural pathways. The current systematic review was conducted to identify whether convergent evidence supporting the usefulness of tDCS to deal with food craving are present in the literature. The review was conducted by searching articles published up to January 1^st^ 2022 on MEDLINE, Scopus and PsycInfo databases. We included studies investigating the effects of tDCS on food craving in subjects affected by overweight and obesity. According to eligibility criteria, 5 articles were included. Results showed that tDCS targeting left DLPFC with unipolar montage induced ameliorating effects on food craving. Controversial results were shown for the other studies, that might be ascribable to the use of bipolar montage, and the choice of other target areas. Further investigations including expectancy effect control, larger sample sizes and follow-up are needed to support more robust conclusions. To conclude, tDCS combined with the use of psychoeducative intervention, diet and physical activity, might represents a potential to manage food craving in individuals with overweight and obesity.

## 1. Introduction

Over the last few decades, obesity has become one of the healthcare-related burdens of the Western society. Indeed, 11% of the European population aged between 18 and 69 and 14% of individuals aged over 65 are affected by this medical condition [1]. Moreover, obesity increases the incidence of diabetes, cardiovascular diseases, and cancer, thus augmenting the cost of the healthcare system, especially for professionals [2]; indeed, among individuals affected by obesity, 31% suffer from hypertension, 8% from diabetes and 8% from respiratory diseases [1]. The prevalence of this condition was particularly exacerbated by COVID-19 quarantine, during which dysfunctional conducts intimately linked to stressful situations such as food craving have dramatically increased [3]–[5]. Food craving is characterized by two intrinsic key-factors: desire intensity for specific food and specificity for certain type of food [6],[7]. Food craving, overeating and erratic alimentary regimen are some of the perpetuating factors of obesity, that can also be ascribable to failure in impulsive behaviour control [8] and dysregulation of many cognitive domains (i.e., decision-making, risk-taking, and working memory). Indeed, this cognitive dysregulation have bidirectionally been associated with obesity and overweight conditions [9] due to the lack of adherence to dietary restrictions and promoting snacking behaviours [10]. Hunger and food intake are regulated by different hierarchical processes that rely on different but intertwined neural circuitries, such as homeostatic and reward circuits, and cognitive areas [11]; in particular, a key area which is primarily involved in modulating impulsiveness and overconsumption is the dorsolateral prefrontal cortex (DLPFC) [11], whose decreased activation has been found in patients with obesity [12], hence preventing the modification of lifestyle habits (e.g., food intake and physical activity) [13]. An impairment in the prefrontal cortical inhibitory networks could be considered as one of the markers of pathophysiology of impulsive behaviour, since the prefrontal cortex plays a pivotal role in decision making and in gating automatic responses [14],[15].

In the context of treatments, tDCS has been revealed to be a promising tool and extensively used in a wide variety of psychiatric and neurological disorders [16]–[19], including food-related dysfunctional behaviour [20]. tDCS involves the modulation of cortical excitability by means of a constant low-amperage electrical current (~ 1–2 milliampere, mA) to the cortex *via* scalp electrodes; cortical excitability is increased by anodic stimulation and decreased by cathodic one [21],[22]. Given the role of the DLPFC in impulsive behaviour control [23],[24], the application of non-invasive brain stimulation techniques has mainly focused on this brain area. In this line, a recent systematic review stated the potential of tDCS on response inhibition, with anodal tDCS over right prefrontal cortex enhancing this cognitive process [25]: this represents an optimal rationale to introduce the use of tDCS in the field of obesity and overweight, given the presence of impulsive behavior and lack of response inhibition.

Since one of the latest reviews on tDCS effects on food craving did not report significant results [26], and this might be ascribable to the inclusion of mixed samples studies, and since the study performed by Mostafavi et al. [27] reported positive effects that might be biased by the inclusion of studies that applied other interventions (i.e., diet, physical exercise) in addition to tDCS, a literature update focusing on a more homogeneous sample and on the mere effect of tDCS is needed. When studying food craving concomitant with other eating disorders such as binge eating and bulimia, results could be biased by the apparent common phenotype of food craving and eating disorder; even though people affected by binge eating and bulimia displayed higher food craving if compared to non-clinical sample [28], there is no compelling evidence that these differences reflect higher reactivity to food cues in terms of larger increases in food craving intensity in response to food cues (which would explain their difficulties in controlling food intake [29].

Our review will focus on the neuro-modulating effects of tDCS on specific brain regions involved in food craving in individuals affected by overweight and obesity.

## 2. Materials and methods

The method used for this systematic review satisfies the Preferred Reporting Items for Systematic Review and Meta-Analysis guidelines (PRISMA), which comprises a checklist to ensure the quality of systematic reviews [30], which is fully reported in the supplementary Materials (Table S1). In order to perform an effective search strategy, we adopted the Population, Intervention, Comparison, Outcomes and Study Design (PICOS) strategy [31] (Table S2).

Our search was conducted up to January 1^st^ 2022, and comprised three main phases: (i) identification: a literature search based on queries on the electronic MEDLINE, Scopus, PsycInfo, databases. Boolean operators “AND” and “OR” were applied to combine the following list of keywords related to tDCS and the keywords related to food craving: “*transcranial direct current stimulation*” AND “*hyperphagia*” OR “*obesity*” OR “*overweight*” OR “*food craving*” OR “*food intake*”; (ii) Screening: a manual screening of the articles yielded during the first phase, by evaluating title and abstract; (iii) Eligibility: a more in-depth assessment of the remaining papers based on full-text reading.

**Figure 1.**
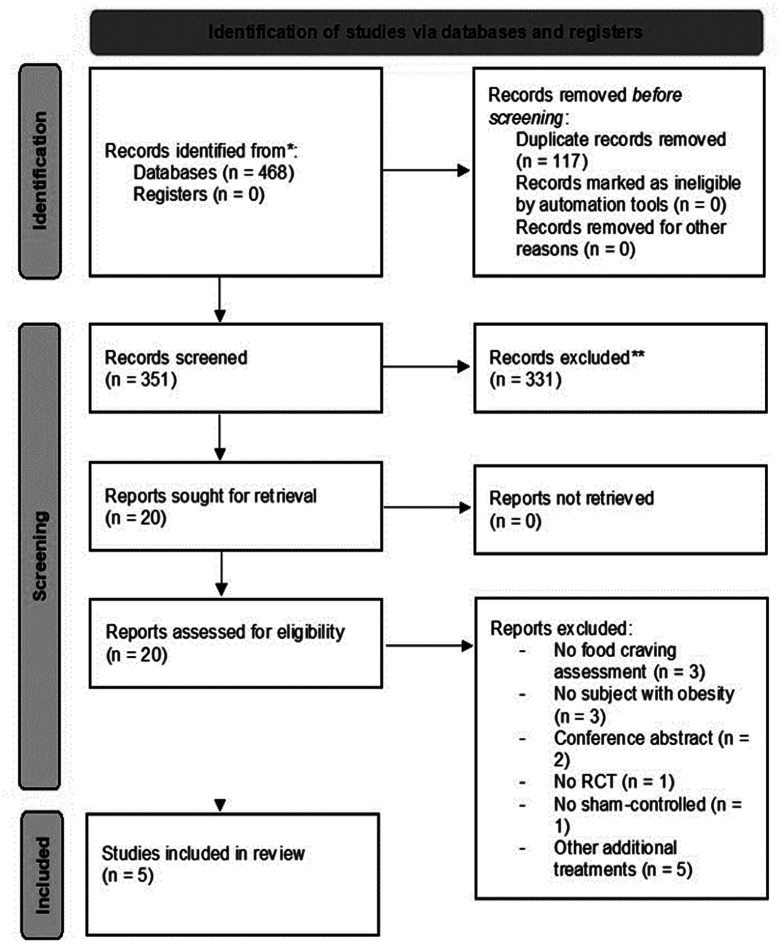
Flow chart of the studies selection.

Only articles in English were included. In addition, we also scanned the references list reported by each study. The selected studies (Figure 1) satisfied the preferred reporting items for systematic Review (PRISMA) [30]. During the selection phase studies were included if the following criteria were met: (1) randomized controlled trials (RCTs); (2) individuals over 18 years old; (3) subjects with overweight (body mass index, BMI, between 25 kg/m^2^ and 29.99 kg/m^2^) or obesity (BMI ≥ 30 kg/m^2^); (5) outcomes for food craving and/or food intake was present; (7) articles published in international peer-reviewed and indexed journals; (8) trials examining tDCS efficacy with sham or control condition; (9) studies including tDCS as the only intervention applied. Studies were excluded if (1) participants were diagnosed with eating disorder or were healthy participants with food craving traits; (2) no sham tDCS (3) protocols with treatments in addition to tDCS; (4) no comparisons between conditions; (5) no food craving assessment; (6) no randomized-controlled, placebo-controlled trials and blinding procedure. Articles retrieved were merged into Mendeley database [32] and duplicates were automatically removed using desktop Mendeley reference manager. For each included study, we computed the effect size of significant results.

To assess the quality of evidence of the studies, we used the modified Jadad scale [33]. The scales ranges from 0 to 8, and points are awarded if the study meets the following qualitative criteria: is described as randomized, 1 point; has appropriate randomization method, 1 point; is described as subject-blinded, 1 point; is described as evaluator-blinded, 1 point; and has description of withdrawals and dropouts, 1 point; presented the inclusion/exclusion criteria, 1 point; described the adverse effects, 1 point; and described statistical analysis, 1 point. The total score for each article was computed by summing the score of each item. Studies with a modified Jadad score ≤ 3 were low-quality randomized controlled trials (RCTs); studies with a modified Jadad score ≥ 4 were considered to be high-quality RCTs. Results of quality assessment are shown in the supplementary materials (Table S3). Also, we used the Cochrane Collaboration's “Risk of Bias” tool to assess bias in randomized controlled studies [34], evaluating the following domains: sequence generation (selection bias), allocation sequence concealment (selection bias), blinding of participants and personnel (performance bias), blinding of outcome assessment (detection bias), incomplete outcome data (attrition bias), selective outcome reporting (reporting bias) and other potential sources of bias. This tool assigns three different score to each analyzed subdomain: low risk, unclear risk, high risk.

Two independent reviewers (E.M. and V.C.) and one referee (G.O.) were involved in all the above-mentioned processes (data sources and search strategy, procedure for studies selection, quality assessment and data extraction). Any disagreements were resolved through consensus of all authors.

## 3. Results

A search of the databases disclosed 468 articles. After duplicates removal, 351 records were screened. After the screening phase, 20 full-text articles were retained. Among these, 14 studies were excluded for the following reasons: 5 of them included other treatments in addition to tDCS; 3 of them did not include food craving/food intake outcomes; 3 studies recruited healthy subjects; 2 studies had abstract only; one study did not perform a randomized design; one study did not perform tDCS sham session. Finally, 5 studies were included (Figure 1).

### 3.1. Characteristics of the studies

#### 3.1.1. Samples

The included studies diverged in sample size dimensions, ranging from a minimum of 9 participants to a maximum of 74 participants. There was a wide sex heterogeneity in the composition of the samples. Four studies included both females and males, while 1 study recruited female participants only [35].

#### 3.1.2. tDCS parameters

Regarding the number of sessions, 2 studies [35],[36] used a single-session protocol, one study [37] performed 2 sessions, one study employed 3 and 6 sessions [38] and one study used 15 sessions [39].

Concerning session duration, 3 studies stimulated for 20 minutes [35]–[37], and 2 studies for 40 minutes [38],[39].

As regards the target area, 2 studies applied a unipolar montage: one of them first administered anodal tDCS (atDCS) and then cathodal tDCS (ctDCS) over the left DLPFC (lDLPFC) [38] and the other one applied anodal tDCS over the lDLPFC [39]; the other 3 studies applied a bipolar montage: two of them stimulated the lDLPFC and the other one stimulated the right DLPFC (rDLPFC) [37].

#### 3.1.3. Experimental design

All the selected studies were randomized and sham controlled, according to inclusion criteria. Concerning the blinding procedure, 2 studies were single blind [37],[39], 2 studies were double blind [35],[38] and one study had a mixed blinding procedure [36]. Regarding the study design, 2 studies were crossover [35],[37], 3 studies were between subjects [36],[38],[39].

#### 3.1.4. Quality assessment of RCTs studies

According to Jadad's score [33], 4 studies out of 5 were high-quality RCTs (score ≥ 4) and one study was classified as nearly high-quality RCTs (score = 3.5). The average between-studies score was 5.5 ± 1.45 (Table S3). For what concern Cochrane Collaboration's “Risk of Bias [34], we reported the labels indicating the risk of bias using different colors (Table S4). The subdomain that resulted to be more at risk is the attrition bias, that indicates incomplete outcome data.

#### 3.1.5. Effect size

The effect size was computed or retrieved from the selected studies to quantify the magnitude of the experimental effect of significance for food craving measures. Since one study reported no significant results [35], we retrieved/computed effect size for a total of 4 studies out of 5. As regards the magnitude of effect size, one study reported a small effect size for food craving parameters—Cohen's d < 0.5 [38]; 2 studies reported an intermediate effect size – 0.5 ≤ Cohen's d < 0.8; 0.060 ≤ partial η2 < 0.11 [36],[37]; 3 studies displayed a large effect size (Cohen's d ≥ 0.8; partial η2 ≥ 0.11) [37]–[39]. To avoid overestimation or underestimation of the results, we stratified effect sizes according to their magnitude (i.e., for each study, different effect sizes belonging to small, medium, or large magnitude might have been reported). The effect sizes were calculated and interpreted using psychometrica software [40], and are reported in Table 1.

**Table 1.** tDCS parameters and outcomes.

Reference	Sample	Study design	Stimulation location	Current intensity/electrode size/duration/ follow-up	Outcome measures	Clinical outcomes and Effect size calculation
Heinitz et al., 2013 [39]	N = 22 subjects with obesity active: n = 9 sham: n = 13	Single-blinded, randomized, parallel design study	a: left DLPFC rc: right supraorbital region	2mA;35 cm^2^; 40 min; 15 sessions, FU: no	Food preferences assessed by SFTTs after vending machine food ad libitum paradigm; VAS for appetite	-Decrease in VAS ratings for hunger (p = 0.01; Cohen's d = 1.149) and urge to eat (p = 0.05; Cohen's d = 0.837) in active group compared to sham group -Decrease of total energy intake during SFTT relatively lower in satiated individuals (p = 0.01; Cohen's d = 1.183)
Gluck et al. 2015 [38]	N = 9 subjects with obesity active: n = 5 sham: n = 4	Randomized, double-blinded, sham-controlled parallel study	Study 1 c: left DLPFC (F3) ra: left forearm Study 2 a: left DLPFC (F3) rc: right eye	Study 1: 2 mA; 25 cm^2^; 40 min; 3 sessions; FU: no Study 2: 2 mA; 25 cm^2^; 40 min; 6 sessions; FU: no	Energy intake and weight change following 3-days ad libitum intake using of an automated vending machines with preferred food	-Reduced consumption of kilocalories per day (p = 0.07), especially for fat food (p = 0.03, Cohen's d = 0.309) and soda (p = 0.02, Cohen's d = 0.334) after atDCS versus ctDCS -Increased % of weight loss (p = 0.009, Cohen's d = 1.09) after atDCS versus ctDCS.
Grundeis et al., 2017 [35]	N = 25 women with obesity	Double-blinded, randomized, sham-controlled, within-subject crossover design	Condition 1: a: left DLPFC; c: right frontal operculum Condition 2: c: left DLPFC a: right frontal operculum	2 mA, 35 cm^2^; 20 min; single session	Self-rated task performed after food picture task; VAS for appetite (baseline, after tDCS and after ad libitum real food buffet)	No effects of atDCS/ctDCS in modulating the desire of visually presented food and calorie intake
Marron et al., 2019 [37]	N = 12 subjects with obesity	Single-blinded, randomized, sham-controlled, crossover study	a: left DLPFC c: right posterior cerebellar lobe	2 mA; 25 cm^2^; 20 min; 2 sessions; FU: no	Food-related cognitive performance (Food-modified N-back task); general effect of motor performance and working memory (Finger tapping task and digit span test); VAS for appetite	-Increased hunger and desire to eat after food exposure in active tDCS and the opposite in sham condition (trend, p = 0.094) -Increased hunger in the active condition compared to baseline (p = 0.019; Cohen's d = -0.79) -Effect of time in desire to eat, indicating an increase for all participants (p = 0.033, ηp_2_ = 0.349) -Increased number of errors in N-back task after active tDCS (trend, p = 0.085) -Improved backward digit span in sham vs active (p = 0.039; Cohen's d = -0.67)
Ray et al., 2019 [36]	N = 74 subjects with obesity and overweight told sham/ got sham: n = 18 told sham /got active: n = 19 told active/got sham: n = 17 told active/ got active: n = 20	Not blind/single blind, randomized design, between-subjects	a: right DLPFC c: left DLPFC	2 mA; 24 cm^2^, single session, 20 min; FU: no	Electronic baseline surveys; Food craving task with highly palatable food; eating task with available real food; hunger assessment	-No main effect of real vs sham tDCS on craving or eating -No interaction effect between tDCS condition and expectation -Less craving and eating in participants who were told receiving active tDCS (p = 0.005, ηp_2_ = 0.09)

*Notes*: a, anodal; atDCS; ctDCS, cathodal tDCS; d, Cohen's effect size; DLPFC, dorsolateral prefrontal cortex; FU, follow up; min, minutes; mA, milliampere; ra, reference anode; rc, reference cathode; SFTTs, snack food taste tests; tDCS, transcranial direct current stimulation; VAS, Visual Analogue Scale; ηp^2^, partial eta square

### 3.2. Food craving outcomes

To investigate possible changes associated with food consumption, Heinitz and colleagues [39] applied short-term and long-term anodal tDCS targeting the lDLPFC. Findings showed that short-term tDCS did not influence ad libitum food consumption from the vending machines and no weight changes were detected after 4 weeks. However, after 6 weeks, participants showed decreased appetite (p = 0.01) and desire to eat (p = 0.05) compared to the sham condition. After long-term active tDCS, satiated subjects displayed less food intake (p = 0.01) compared to the sham group. The same target area was also chosen by Gluck et al. [38] in the two following protocols: a) ctDCS over the lDLPFC or sham tDCS (study 1); and b) atDCS over the lDLPFC or sham (study 2); participants were exposed to automatic vending machines with their preferred food on three consecutive mornings in both studies. Based on their results, participants showed an overall reduction of calories intake (p = 0.07) per day, accompanied by a significant weight loss (p = 0.02) after atDCS compared to ctDCS.

No significant effects were detected by Grundeis and colleagues [35], neither after atDCS over the lDLPFC, nor after ctDCS over the lDLPFC on food intake and desire generated by food pictures.

Another study investigating atDCS and ctDCS over the DLPFC was carried out by Ray et al. [36]. In their study, atDCS and ctDCS were delivered over the rDLPFC and lDLPFC, respectively. Only two groups out of four were blinded for tDCS application (sham/active condition). One group was told it would receive sham tDCS and instead it underwent active tDCS, while the other blind group was told the opposite. Participants who were told they would receive active tDCS reported decreased craving and eating (p < 0.01), compared to those who were told they would receive sham, regardless of real condition.

Differently from the aforementioned studies, Marron and colleagues [37] explored the effects of modulating DLPFC-cerebellum interactions by applying a new two-session tDCS paradigm on subjects with obesity. The study showed a nearly significant decrease of hunger and desire to eat after sham tDCS measured by VAS (p = 0.094), whereas active condition did not (p = 0.903). Additionally, after active tDCS, subjects showed an increased number of errors during the food-modified working memory task compared to sham condition. Participants also displayed higher scores in the working memory task (Backward Digit Span Test) after the second session in both conditions; sham condition improved backward digit span compared to the active condition.

## 4. Discussion

This review provided a qualitative synthesis of the effects of tDCS on food craving in subjects affected by obesity and overweight, taking into account several tDCS parameters (i.e., targeted areas, number of sessions), and the magnitude of the experimental effects.

All studies targeted DLPFC, but differences were detected for the lateralization of the stimulation site and montages (e.g., extracephalic or cephalic return). DLPFC was the sole target area in all the analyzed studies, and this is in line with the pivotal role of DLPFC in executive functions (e.g., planning, decision making, response inhibition and goal-directed behaviour) [41].

Coherently with the hypothesis explaining the pathogenetic mechanism that might sustain food craving behaviour in obesity, the findings of the current work have been reported according to DLPFC lateralization. According to the right brain hypothesis of obesity [42] postulating rDLPFC lateralization for hunger and eating behaviour, the enhancement of its activity could strengthen inhibitory control by orexigenic areas to suppress hunger in overeating subjects. The pivotal role of the rDLPFC is consistent with recent findings showing altered between-network connectivity in the basal ganglia and rDLPFC in subjects predisposed to obesity, that might reflect a tendency towards habitual behaviour rather than goal-directed behaviour in these individuals [41].

The second hypothesis arose from studies that reported decreased grey matter density in the lDLPFC [43] and lower activation in the same area after food intake in subjects with obesity. as compared to subjects with BMI within normative range in resting state condition [11],[44]. Ray and colleagues [36] applied atDCS over the rDLPFC and ctDCS over the lDLPFC, but the reduction of food craving was mainly sustained by placebo effect, indicating that expectancy effect was more powerful than the main effect of tDCS itself. In fact, since placebo effect derives from subjective interpretation of context information and most of medical treatments benefits are caused by the brain's response to the treatment context [45], the sole sight of tDCS montage might have acted as contextual cue inducing an expectancy effect in subjects, and it is possible to argue that tDCS might elicit strong behavioural effects regardless of the condition [46]. A recent study found that current intensities conventionally used in non-invasive brain stimulation techniques studies are likely to be insufficient to affect neuronal circuits in a direct way, suggesting that the reported behavioural and cognitive effects may result from indirect mechanisms [41].

The role of lDLPFC has been reported to be essential also for dietary self-control. In fact, during decisions about food preference, subjects with higher self-control displayed increased activity in this area. Therefore, differences in DLPFC activity, particularly the lDLPFC, may explain individual differences in dietary choices, “*vis-à-vis the connection between the DLPFC and inhibitory control*” [27]. Differently, Grundeis and colleagues [35] applied atDCS over the lDLPFC and ctDCS over the right frontal operculum and vice versa, due to its involvement in gustatory processes [47] and its higher activation during food desire regulation in subjects affected by obesity [48]. Marron and colleagues [37] stimulated the lDLPFC with anode and the right posterior cerebellar lobe with cathode, as the cerebellum is one of the targets of leptin hormone [49] and it is activated by food cues [50]. These last two studies did not find the expected results, and Marron and colleagues [37] even reported an increase in hunger after active tDCS. As explained by authors, this unexpected result may originate from a functional decoupling between the lDLPFC and the cerebellum. In addition, the correlation between decreased activity in the cerebellum and increased hunger is consistent with the inverse relationship between cerebellar integrity and BMI [51].

As regards the comparison between tDCS parameters, the studies performed by Gluck et al. [38] and Grundeis et al. [35] adopted different montages and polarities to detect differences in food craving outcomes. Gluck et al. [38] found that atDCS exerted a significant ameliorating effect as compared to ctDCS. These differences might be the result of neuronal depolarization induced by anodal stimulation. Furthermore, it is worth noting that Grundeis et al. [35] recruited female participants with obesity, and they did not inquire the menstrual cycle; in this context, the menstrual cycle has been reported to be likely to affect food perception and intake [52].

Considering the montage, Gluck et al. [38] and Heinitz et al. [39] found out a decrease in food craving, and it could be suggested that the use of extracephalic reference cathode might exert a deeper stimulation allowing a more powerful effect. This is also in line with the aforementioned theory of the lDLPFC role in regulating food craving [11],[44].

Furthermore, tDCS montage could have acted as a confounding variable, since tDCS induces its highest and longest-lasting EEG changes when delivered bipolarly with the anode on the left and the cathode on the right prefrontal cortex, and unipolarly with the anode on the right prefrontal cortex [53]. The depth of stimulation when using extracephalic cathode location is substantially greater than that of the traditional cephalic arrangement. This would indicate that a higher percentage of the desired stimulation area would be covered through extracephalic locations. Investigation of additional cut planes confirms these results [54].

It is worth noting that all the included studies showed no effect ascribable to tDCS current strength and duration or number of sessions. This result is consistent with recent findings in electrical stimulation literature showing no significant difference in cortical excitability induced by these parameters, which might depend on the high degree of inter-subject variability in the neurophysiological response to tDCS [26]. However, the reported decreased excitability produced by long duration of tDCS [55] might be referred to phenomena of neuronal habituation.

The current work has some limitations that need to be addressed. First of all, the small pool of included studies (N = 5) impede robust qualitative conclusions. Other issues concern the heterogeneity of included studies, that prevent a meta-analytic comparison, too.

Additionally, the computation of the magnitude of the effects on food craving measures prevented robust conclusions, since most of the studies analyzed reported small or medium effects sizes.

In addition, an analysis of potential confounders has been conducted to disentangle the issues of mixed results (Table 2).

**Table 2.** Possible confounding factors affecting the direction of results.

Sample	Type of design	Blinding procedures	Stimulation location	Number of sessions and duration	Outcome measures
- Unbalanced distribution of female and male within studies	- Crossover VS between subject VS within subject	- Single blinded VS double blinded	- Active stimulation: left vs right side;	- Single session VS repeated sessions	- Food craving outcomes: behavioral VS self-report measures
			- Return electrode: cephalic vs extracephalic	- Duration: 20 min VS 40 min	

## 5. Conclusions

In conclusion, the revised studies showed controversial results. Indeed, it is not possible to discern whether positive effects can be ascribable to expectancy effects or to tDCS effect itself. Therefore, future tDCS protocols, including expectancy effect control, should be adopted. As regards the target area, clarifying a possible lateralization in food intake regulation and comparing the anodal stimulation of both right and left DLPFC in the same study should be recommended. It is worth to note that tDCS has some technical limitations such as low spatial accuracy that prevent an optimal target precision (the current passes through the brain from anode to cathode and modulates neural activity simultaneously underneath anode and cathode, and it might be hard to associate the effects of tDCS to a specific brain area). In addition, long-term effect after tDCS use is not in depth investigated, and so not well established [56]. Lastly, the difference between active and sham session might be statistically but non clinically significant [57].

tDCS technique is broadly safe, and does not cause permanent or severe damage or discomfort when used according to the safety guidelines. However, several risks have been reported, and they might represent therapeutic limitations. In fact, sensations such as tingling, itching, and burning sensations under the electrodes, mild headaches, fatigue, reported by individuals during and/or after the administration of tDCS [58] can decrease individuals' adherence to protocols, thus preventing therapeutic benefits. On the other hands, the increase of individuals' adherence might also represent the therapeutic goal of tDCS: for example, some studies reported an increased therapeutic adherence to treatments following tDCS, thus helping the self-management of individuals' health [59],[60].

For the aforementioned reasons, in the field of obesity and overweight, it is possible to program personalized intervention to reduce weight and enhance subjects' quality of life with a more holistic intervention, as tDCS alone might not be sufficient to reach this aim. In fact, tDCS, combined with the use of psychoeducative intervention, diet and physical activity, might represents a potential to manage food craving in individuals with overweight and obesity.
