# Supplementary material for: The effects of Transcranial Direct Current Stimulation on food craving and food intake in individuals affected by obesity and overweight: a mini review of the magnitude of the effects
Source: AIMS Neurosci. 2022 Aug 1;9(3):358–72. doi: 10.3934/Neuroscience.2022020 (PMC9581736; doi:10.3934/Neuroscience.2022020)
Supplement: Supplementary file 1 [file neurosci-09-03-020-s001.pdf]

Neuroscience

AIMS Neuroscience, 9(3): 358–372.
DOI: 10.3934/Neuroscience.2022020
Received: 15 March 2022
Revised: 14 July 2022
Accepted: 20 July 2022
Published: 01 August 2022

http://www.aimspress.com/journal/neuroscience

Mini review

The effects of Transcranial Direct Current Stimulation on food craving and food intake in individuals affected by obesity and overweight: a mini review of the magnitude of the effects

Graziella Orrù[1,*], Valentina Cesari[1], Eleonora Malloggi[1], Ciro Conversano[1], Danilo Menicucci[1], Alessandro Rotondo[2], Cristina Scarpazza[3,4,5], Laura Marchi[1] and Angelo Gemignani[1]

[1] Department of Surgical, Medical and Molecular Pathology and Critical Care Medicine, University of Pisa, via Savi, 10, 56126, Pisa, Italy
[2] Department of Law, Criminal Law, University of Pisa, via Curtatone e Montanara, 15, 56126, Pisa, Italy
[3] Department of General Psychology, University of Padova, Via Venezia 8, Padova, 35131, Italy
[4] IRCCS S Camillo Hospital, Via Alberoni 70, 30126 Venezia, Italy
[5] Padova Neuroscience Centre, University of Padova, Via Giuseppe Orus 2, 35131 Padova, Italy

* **Correspondence:** Email: graziella.orru@unipi.it.

Supplementary files

Table S1. Prisma Checklist.

Section/topic	#	Checklist item	Reported on page #
**TITLE**			
Title	1	Identify the report as a systematic review, meta-analysis, or both.	1
**ABSTRACT**			
Structured summary	2	Provide a structured summary including, as applicable: background; objectives; data sources; study eligibility criteria, participants, and interventions; study appraisal and synthesis methods; results; limitations; conclusions and implications of key findings; systematic review registration number.	1-2
**INTRODUCTION**			
Rationale	3	Describe the rationale for the review in the context of what is already known.	3
Objectives	4	Provide an explicit statement of questions being addressed with reference to participants, interventions, comparisons, outcomes, and study design (PICOS).	3
**METHODS**			
Protocol and registration	5	Indicate if a review protocol exists, if and where it can be accessed (e.g., Web address), and, if available, provide registration information including registration number.	NA
Eligibility criteria	6	Specify study characteristics (e.g., PICOS, length of follow-up) and report characteristics (e.g., years considered, language, publication status) used as criteria for eligibility, giving rationale.	3-4, Table S2
Information sources	7	Describe all information sources (e.g., databases with dates of coverage, contact with study authors to identify additional studies) in the search and date last searched.	3-4

Search	8	Present full electronic search strategy for at least one database, including any limits used, such that it could be repeated.	3
Study selection	9	State the process for selecting studies (i.e., screening, eligibility, included in systematic review, and, if applicable, included in the meta-analysis).	3-4
Data collection process	10	Describe method of data extraction from reports (e.g., piloted forms, independently, in duplicate) and any processes for obtaining and confirming data from investigators.	4
Data items	11	List and define all variables for which data were sought (e.g., PICOS, funding sources) and any assumptions and simplifications made.	Table S2
Risk of bias in individual studies	12	Describe methods used for assessing risk of bias of individual studies (including specification of whether this was done at the study or outcome level), and how this information is to be used in any data synthesis.	Table S3, Table S4, Table 2
Summary measures	13	State the principal summary measures (e.g., risk ratio, difference in means).	Table 1
Synthesis of results	14	Describe the methods of handling data and combining results of studies, if done, including measures of consistency (e.g., I^2) for each meta-analysis.	NA

**Section/topic**	**#**	**Checklist item**	**Reported on page #**
Risk of bias across studies	15	Specify any assessment of risk of bias that may affect the cumulative evidence (e.g., publication bias, selective reporting within studies).	4, Table S3, Table S4
Additional analyses	16	Describe methods of additional analyses (e.g., sensitivity or subgroup analyses, meta-regression), if done, indicating which were pre-specified.	NA
**RESULTS**			
Study selection	17	Give numbers of studies screened, assessed for eligibility, and included in the review, with reasons for exclusions at each stage, ideally with a flow diagram.	4, Figure 1

Study characteristics	18	For each study, present characteristics for which data were extracted (e.g., study size, PICOS, follow-up period) and provide the citations.	5-9, Table 1
Risk of bias within studies	19	Present data on risk of bias of each study and, if available, any outcome level assessment (see item 12).	6, Table S3, Table S4
Results of individual studies	20	For all outcomes considered (benefits or harms), present, for each study: (a) simple summary data for each intervention group (b) effect estimates and confidence intervals, ideally with a forest plot.	5-9, Table 1
Synthesis of results	21	Present results of each meta-analysis done, including confidence intervals and measures of consistency.	NA
Risk of bias across studies	22	Present results of any assessment of risk of bias across studies (see Item 15).	6, Table S3, Table S4
Additional analysis	23	Give results of additional analyses, if done (e.g., sensitivity or subgroup analyses, meta-regression [see Item 16]).	NA
**DISCUSSION**			
Summary of evidence	24	Summarize the main findings including the strength of evidence for each main outcome; consider their relevance to key groups (e.g., healthcare providers, users, and policy makers).	9-11
Limitations	25	Discuss limitations at study and outcome level (e.g., risk of bias), and at review-level (e.g., incomplete retrieval of identified research, reporting bias).	11-12, Table S3, Table S4, Table 2
Conclusions	26	Provide a general interpretation of the results in the context of other evidence, and implications for future research.	11-12
**FUNDING**			
Funding	27	Describe sources of funding for the systematic review and other support (e.g., supply of data); role of funders for the systematic review.	NA

Table S2. Population, Intervention, Comparison, Outcomes and Study Design (PICOS).

**Parameter**	**Inclusion criteria**	**Exclusion criteria**
Population	Participant over 18 years old;) subjects with overweight (body mass index, BMI, between 25 kg/m^2 and 29.99 kg/m^2) or obesity (BMI ≥ 30 kg/m2);	Participants with diagnosis of eating disorders (bulimia or BED); healthy subjects with food craving and non-pathological binge eating behaviour
Interventions	Experimental procedure comprising usage of active tDCS and sham laboratory-controlled tDCS.	Paradigms not using sham-controlled tDCS; paradigms using home-based tDCS;) protocols with treatments in addition to tDCS;
Comparisons	Participants undergoing to active tDCS vs sham tDCS; participants undergoing to anodal tDCS vs cathodal tDCS vs sham tDCS	No comparisons between conditions (to anodal tDCS vs cathodal tDCS vs sham tDCS) no within subject design) or groups (no between subjects design; active group versus sham group)
Outcomes	Clinical, behavioral and physiological outcomes for food craving and/or food intake;	Outcomes not assessing food craving or food intake
Study design	Between subjects; crossover; within subjects; randomized controlled; placebo controlled; single-blind; double blind	no randomized-controlled, placebo-controlled trials and blinding procedure

Table S3. Modified Jadad Scale for Quality assessment of RCTs.

Author and Year	Was the study described as randomised?	Was the method of randomization appropriate?	Was the study described as blinded? (double-blind with score 1; single-blind with score 0.5)	Was the method of blinding appropriate?	Was there a description of withdrawals and dropouts?	Was there a clear description of the inclusion/exclusion criteria?	Was the method used to assess adverse effects described?	Was the method of statistical analysis described?	**JADAD SCORE**
Heinitz et al., 2013 [39]	1	1	0.5	0	1	1	1	1	6.5
Gluck et al. 2015 [38]	1	1	1	1	0	**1**	1	1	7
Grundeis et al., 2017 [35]	1	0	1	1	0	1	1	1	6
Marron et al., 2019 [37]	1	0	0.5	0	0	1	1	1	4.5
Ray et al., 2019 [36]	1	0	0.5	0	0	1	0	1	3.5

1= Yes; 0=No; 0= Not described; 1 =double blind 0.5=single blind

Table S4. Cochrane Collaboration's Risk of Bias.

Study	Selection bias		Reporting bias	Other bias	Performance bias	Detection bias	Attrition bias
	Random sequence generation	Allocation concealment					
Heinitz et al., 2013 [39]	🟢	🔴	🟢	🟢	🔴	🔴	🟢
Gluck et al., 2015 [38]	🟢	🟢	🔴	🟢	🟢	🟢	🔴
Grundeis et al., 2017 [35]	🔴	🟢	🟢	🟢	🟢	🟢	🔴
Marron et al., 2019 [37]	🔴	🔴	🟢	🟡	🔴	🔴	🔴
Ray et al., 2019 [36]	🔴	🔴	🟢	🟡	🟢	🟢	🔴

🟢 Low risk

🔴 High risk

🟡 Unclear risk

AIMS Press

© 2022 the Author(s), licensee AIMS Press. This is an open access article distributed under the terms of the Creative Commons Attribution License (http://creativecommons.org/licenses/by/4.0)
